# Multi-omics characterization of C4orf19 in HNSCC: constructing prognostic signatures for immunotherapy and chemotherapy response prediction

**DOI:** 10.1186/s12885-026-15633-y

**Published:** 2026-01-30

**Authors:** Da Huang, Yuxuan Guo, Mingfen Chen, Guoqun Chen, Guangchun He, Chanjuan Zheng, Shujun Fu, Jie Wang, Yian Wang, Xiyun Deng

**Affiliations:** 1 Changsha Hospital of Hunan Normal University (The Fourth Hospital of Changsha), Changsha, 410006 China; 2 Department of Pathophysiology, Key Laboratory of Model Animals and Stem Cell Biology in Hunan Province, Hunan Normal University Health Science Center, No. 371, Tongzipo Road, Yuelu District, Changsha, 410013 China; 3 Department of Pathology, Institute of Oncology, The School of Basic Medical Sciences & Diagnostic Pathology Center, Fujian Medical University, Fuzhou, 350122 China; 4 Department of Radiation Oncology, The Second Affiliated Hospital of Fujian Medical University, Fujian Medical University, Quanzhou, 362000 China

**Keywords:** Head and neck squamous cell carcinoma, C4orf19, Prognostic biomarker, 5-FU chemosensitization, Immunotherapy response prediction

## Abstract

**Backgrounds:**

C4orf19, a protein encoded by an open reading frame on human chromosome 4, exhibits aberrant expression in various tumors and is linked to patient prognosis, though its role in head and neck squamous cell carcinoma (HNSCC) remains unclear.

**Methods:**

TCGA and GEO datasets were analyzed to evaluate the prognostic value of C4orf19 in HNSCC. Functional assays (EdU, immunofluorescence, and CCK-8) were used to assess the effects of C4orf19 knockdown on tumor cell proliferation. Drug sensitivity analyses using the GDSC database and 5-fluorouracil (5-FU) chemosensitization experiments were performed. Bioinformatics approaches (CIBERSORT, ssGSEA, xCell, and maftools) were applied to explore associations between C4orf19 expression, the tumor microenvironment (TME), immune checkpoints, and tumor mutational burden (TMB).

**Results:**

C4orf19 was downregulated in HNSCC patients and was associated with a poor prognosis. While C4orf19 knockdown did not affect proliferation of HNSCC cells, it enhanced 5-FU sensitivity. Mechanistically, C4orf19 may influence immunotherapy efficacy by modulating immune checkpoint gene expression, remodeling TME, and regulating TMB. These findings suggest that C4orf19 expression may serve as a predictive biomarker for immunotherapy response in HNSCC patients.

**Conclusions:**

C4orf19 expression shows potential as a predictive biomarker for immunotherapy response in HNSCC patients. The study highlights its role in enhancing chemosensitization and predicting immunotherapy efficacy, positioning C4orf19 as a promising prognostic biomarker and therapeutic predictor in HNSCC.

**Information:**

The online version contains supplementary material available at 10.1186/s12885-026-15633-y.

## Introduction

Head and neck squamous cell carcinoma (HNSCC) originates from the squamous epithelium of the oral cavity, pharynx, and larynx and is one of the most common malignant tumors in the head and neck region [1]. In 2020, there were over 870, 000 newly diagnosed cases and more than 440, 000 deaths worldwide attributed to HNSCC [2]. The incidence of HNSCC continues to rise globally and is projected to increase by 30% by 2030, reaching 1.08 million annual new cases [3]. Major risk factors for HNSCC include smoking, alcohol consumption, betel nut chewing, and human papillomavirus (HPV) infection [4]. Current standard treatment for HNSCC involves surgery combined with radiotherapy and chemotherapy [5]. While this approach is effective for early-stage disease and may be curative, it provides only palliative benefits for advanced-stage patients [6]. The development of drug resistance frequently results in poor treatment outcomes. The current 5-year survival rate for patients with HNSCC is approximately 60% [7]. Although EGFR-targeted therapy [8] and immunotherapy [9–11] have advanced HNSCC treatment, only a subset of patients benefits from these modalities. The absence of specific predictive biomarkers for treatment selection remains a critical limitation in clinical management. Therefore, identifying novel biomarkers to enable precision therapy, enhance chemosensitivity, and improve HNSCC outcomes represents an urgent unmet need.

Chromosome 4 Open Reading Frame 19 (C4orf19) is a nuclear-cytoplasmic protein encoded by a gene on human chromosome 4. Although its biological functions remain incompletely characterized, emerging evidence suggests its involvement in cancer pathogenesis and metabolism [12]. Recent studies demonstrated that high C4orf19 expression correlates with better prognoses and enhanced immune function in clear cell renal cell carcinoma [13], while exerting tumor-suppressive effects in colon cancer and triple-negative breast cancer [12, 14]. These findings imply a complex, context-dependent role of C4orf19 in oncology. Currently, no studies have investigated C4orf19 in other malignancies, and its function in HNSCC has not been explored.

In this study, we employed bioinformatics analysis to demonstrate that C4orf19 is downregulated in HNSCC and correlates with favorable prognosis. Notably, elevated C4orf19 expression potentiates 5-FU sensitivity in HNSCC cells. Integrative analyses revealed significant associations between C4orf19 expression and immune checkpoint molecules, tumor microenvironment (TME) composition, and tumor mutation burden (TMB), suggesting its utility as a predictive biomarker for immunotherapy response. Collectively, our findings position C4orf19 as a clinically actionable biomarker for guiding chemotherapy and predicting immunotherapy efficacy in HNSCC patients.

## Materials and methods

### Clinical sample

This study used biopsy specimens from 30 treatment-naive HNSCC patients (no prior radiotherapy, chemotherapy, targeted therapy, or immunotherapy) who underwent surgery at Changsha Hospital of Hunan Normal University (The Fourth Hospital of Changsha) between January 2020 and June 2024. All diagnoses were pathologically confirmed. Specimen collection and data acquisition were approved by institutional ethics, with written informed consent from all participants.

### Cell culture

Oral squamous cell carcinoma cell lines Cal-27 and SCC-9 were kindly provided by Hunan Cancer Hospital and authenticated via mycoplasma testing and STR profiling. They were cultured in DMEM (PM150210, Pricella) and DMEM/F12 (PM150312, Pricella) respectively, supplemented with 10% fetal bovine serum (F101-01, Vazyme) at 37 °C and 5% CO₂.

### Data preparation

HNSCC and normal head and neck epithelial tissue RNA-seq data were downloaded from TCGA on June 20, 2024. Clinical data came from UCSC Xena (https://xena.ucsc.edu/). Post-normalization, 515 HNSCC and 44 normal samples were analyzed.

GSE30784 and GSE41613 datasets were obtained from GEO (https://www.ncbi.nlm.nih.gov) on September 13, 2024. After data normalization and clinical info organization, GSE30784 included 167 HNSCC patients and 45 normal samples, while GSE41613 contained 97 HNSCC cases.

### Prognostic analysis

Kaplan-Meier (KM) survival curves were generated with the R “survival” package to assess the association between C4orf19 expression and overall survival (OS) in HNSCC patients. Univariate and multivariate Cox regression analyses (via the “survival” package) incorporated C4orf19 expression and relevant clinical parameters; variables with *p* < 0.05 were considered independent prognostic factors. A nomogram integrating C4orf19 expression and key clinicopathological variables predicted HNSCC outcomes. Calibration and receiver operating characteristic (ROC) curves evaluated nomogram performance.

### Functional enrichment analysis

TCGA-HNSC data were grouped by median C4orf19 expression. Differentially expressed genes (DEGs) were identified via the R package “DESeq2”. Functional annotation of DEG sets included Gene Ontology (GO) and Kyoto Encyclopedia of Genes and Genomes (KEGG) pathway enrichment using the “clusterProfiler” R package. Gene Set Enrichment Analysis (GSEA) with MSigDB hallmark gene sets identified significantly enriched pathways.

### Drug sensitivity analysis

Drug response data were obtained from the Genomics of Drug Sensitivity in Cancer (GDSC) database (https://www.cancerrxgene.org/). Responses of TCGA-HNSC patients to 367 GDSC compounds were analyzed via the R package “oncoPredict”, with drug sensitivity evaluated using IC50-like scores.

### Tumor microenvironment analysis

Proportions of 22 immune cell types in HNSCC TME were quantified via R package “CIBERSORT”, and 28 immune cell infiltration levels analyzed with “ssGSEA”. “Xcell” evaluated immune cell proportions and relative abundances. Pearson correlation analysis assessed correlations between C4orf19 expression, immune cell infiltration, and immune checkpoint levels, visualized via scatter plots.

### Tumor mutation burden analysis

Somatic mutation data were obtained from TCGA-HNSC. Recurrently mutated genes and their variant classifications in HNSCC patients were visualized via the R package “maftools”.

### Immunohistochemistry (IHC)

IHC staining was performed on 30 HNSCC samples and 10 adjacent normal squamous epithelium specimens. A semi-quantitative H-score (staining intensity [0–3] × positive cell percentage [0–100%], range 0–12) was independently assessed by two pathologists. Antibody details are in Supplementary Table 1.

### Plasmids, siRNAs, and transfection methods

Plasmid Construction: Human C4orf19 overexpression plasmid was from CNS Biotechnology (Hunan, China).

siRNA Transfection: Three specific siRNAs and a negative control were designed and synthesized by GenePharma Co., Ltd (Suzhou, China); sequences are in Supplementary Table 2.

Cell Transfection: Cells in logarithmic growth phase were seeded into 6-well plates (CCP-6 H, Servicebio). Transfection was done with Beyotight™ Lipo8000 Transfection Reagent per manufacturer’s protocol when cells reached ~ 60% confluence, then incubated at 37 °C with 5% CO₂ for 48 h.

### Western blot

All experiments followed the method in reference [15]. Antibody details are in Supplementary Table 1.

### Edu assay

Transfected cells were seeded into 12-well plates (3513, Corning). After 24h, EdU assay was done per manufacturer’s protocol (ATXG18171, Abbkine).

### Immunofluorescence (IF)

At 24h post-transfection, cells in 12-well plates were fixed with 4% PFA for 30 min at room temperature, permeabilized with 0.1% Triton X-100 for 20 min, and blocked with 3% BSA for 1 h. Primary antibodies were incubated overnight at 4 °C in the dark. After three 5-min TBST washes, secondary antibodies were incubated for 1 h in the dark. Nuclei were counterstained with DAPI for 5 min, and images were captured using a fluorescence microscope (EVOS M5000, Thermo Fisher Scientific). Antibody details are in Supplementary Table 1.

### Cell counting kit‑8 (CCK-8) assay

Post-transfection, cells were seeded into 96-well plates (CCP06-096, BIOLAND) and treated with 5-FU culture medium at varying concentrations. After 48 h incubation, CCK-8 reagent (GK10001, GLPBio) was added, and absorbance was measured at 450 nm via microplate reader (Synergy 2, Biotek).

### Colony formation assay

Post-transfection, cells were seeded into 12-well plates (3513, Corning). After 48 h, they were treated with 5-FU culture medium at varying concentrations. After 10 days, colonies were fixed with methanol, stained with 0.1% crystal violet, and quantified.

### Data analysis and statistic

Statistical analyses used R (v4.4.1). All results are presented as means ± standard deviations (SD) of at least three independent experiments. Two-group differences were analyzed by unpaired Student’s t-test or Wilcoxon test. *p* < 0.05 was significant.

## Results

### C4orf19 is downregulated in HNSCC

**Fig. 1:**
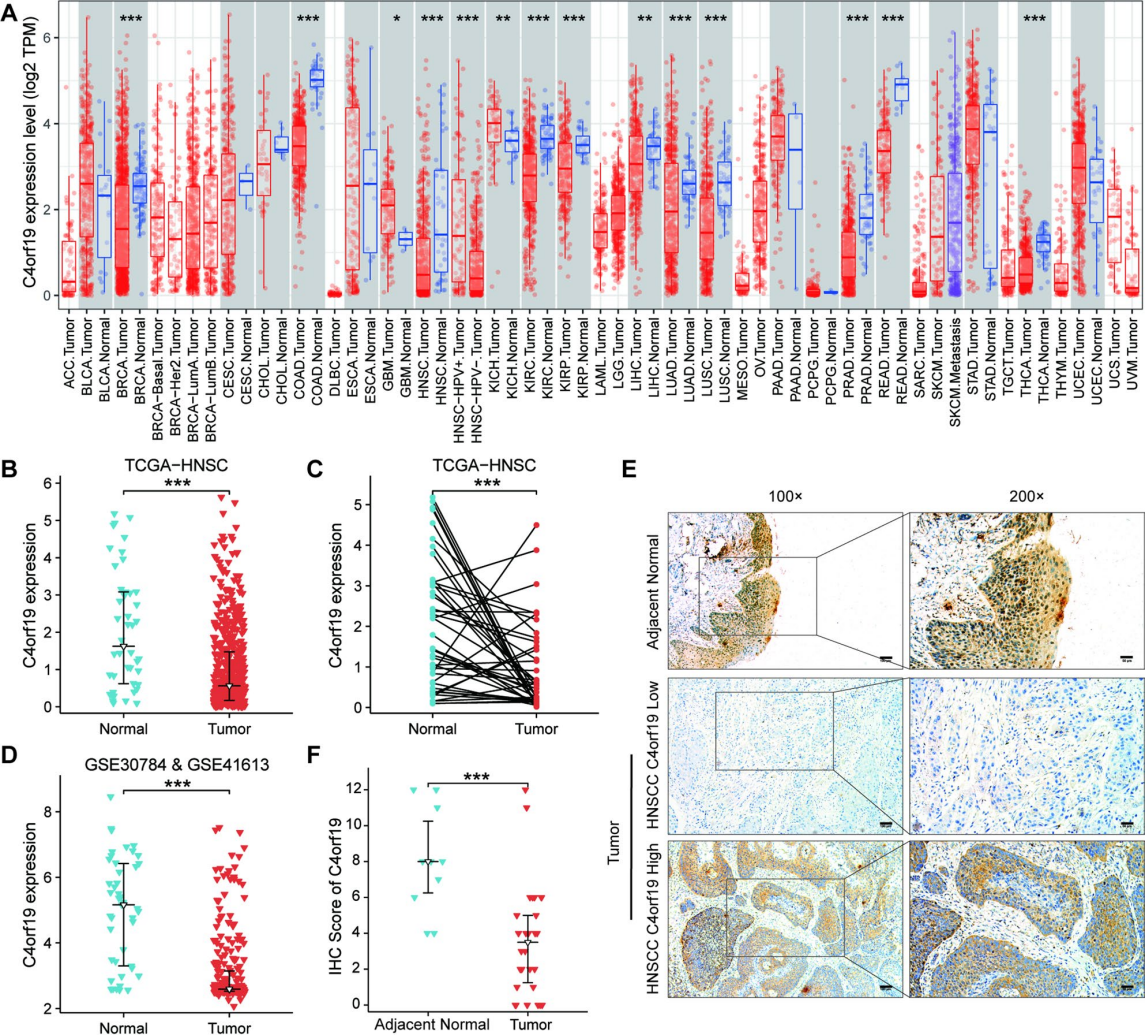
Downregulation of C4orf19 in HNSCC Patients. **A** C4orf19 expression across 33 tumor types compared to normal tissues in the TCGA database. **B, C** C4orf19 expression in HNSCC tumor tissues (n = 515) and adjacent normal tissues (n = 44) (B), as well as in matched tumor tissues (n = 44) and adjacent normal tissues (n = 44) (C) from the TCGA-HNSC dataset. **D** C4orf19 expression in HNSCC tumor tissues (n = 264) and normal tissues (n = 45) after merging the GSE30784 and GSE41613 datasets. **E, F** IHC staining of C4orf19 expression in paraffin-embedded HNSCC tissue (n = 30). Representative images are shown in (E), and quantification of immunohistochemical scores are presented in (F). (Adjacent Normal, n = 10; Tumor, n = 30) (*, *p* < 0.05; **, *p* < 0.01; ***, *p* < 0.001)

To investigate the differential expression of C4orf19 in tumor tissues, we visualized C4orf19 expression across pan-cancer datasets using the TIMER database. Our results demonstrated that C4orf19 was downregulated in multiple cancers including breast cancer, colorectal cancer, HNSCC, kidney renal clear cell carcinoma, kidney renal papillary cell carcinoma, liver hepatocellular carcinoma, lung adenocarcinoma, lung squamous cell carcinoma, prostate adenocarcinoma, rectal adenocarcinoma, and thyroid carcinoma, but upregulated in glioblastoma multiforme and kidney chromophobe (Fig. 1A). We focused on C4orf19 expression in HNSCC. Analysis of tumor-normal paired samples from TCGA-HNSC revealed significant downregulated of C4orf19 in tumor tissues compared with adjacent normal tissues (Fig. 1B, C). For further validation, we analyzed HNSCC Affymetrix microarray datasets (GSE30784 and GSE41613) from the GEO database. After batch-effect correction and data integration, the combined analysis confirmed decreased C4orf19 expression in HNSCC tissues (Fig. 1D). Additionally, IHC staining of paraffin-embedded tissue from 30 HNSCC patients showed significantly lower C4orf19 protein levels in tumors than in adjacent normal epithelium (Fig. 1E, F). Taken together, these results demonstrate consistent downregulation of C4orf19 in HNSCC across multiple platforms.

### Low expression of C4orf19 is associated with poor prognosis in HNSCC patients

**Fig. 2:**
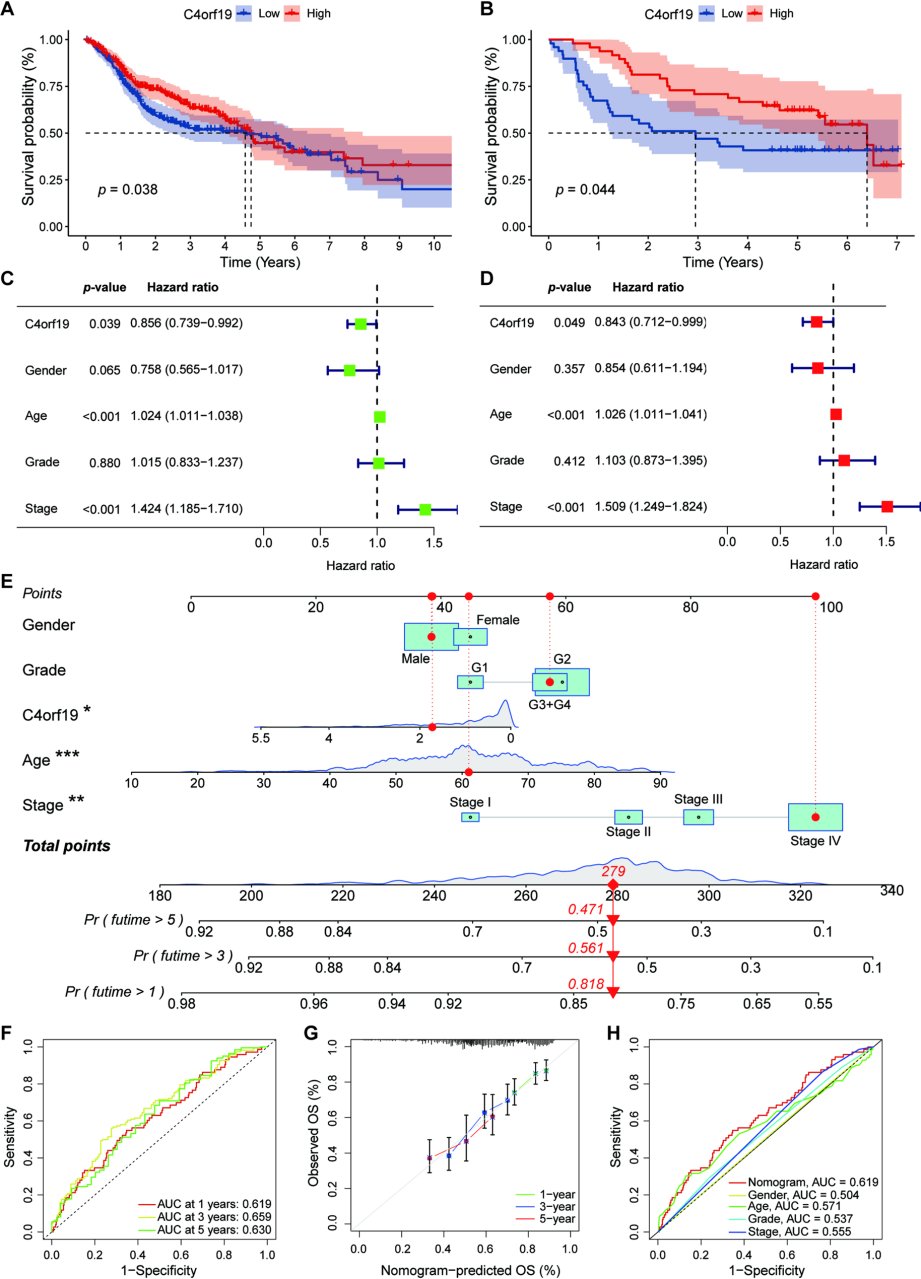
Association between C4orf19 expression and prognosis in HNSCC patients. **A, B** KM survival analysis of C4orf19 expression in TCGA-HNSC (A) and GSE41613 (B) cohorts. **C, D** Univariate (C) and multivariate (D) Cox regression analyses of C4orf19 expression and clinical characteristics in TCGA-HNSC. **E** Nomogram integrating C4orf19 expression and clinical features for survival prediction. **F** Time-dependent ROC curves evaluating the nomogram's predictive accuracy at 1-, 3-, and 5-year intervals. **G** Calibration plots assessing the nomogram's performance at 1, 3, and 5 years. **H** ROC analysis comparing the prognostic predictive power of the nomogram versus individual clinical features

To evaluate the prognostic significance of C4orf19 in HNSCC, we analyzed survival data from the TCGA-HNSC database and GSE41613 dataset. Patients were stratified into high- and low-expression groups based on median C4orf19 expression. KM survival analysis demonstrated that higher C4orf19 expression was associated with improved OS in both cohorts (Fig. 2A, B). Univariate and multivariate cox regression analyses identified C4orf19 expression, age, and tumor stage as independent prognostic factors for OS (Fig. 2C, D). We developed a nomogram incorporating C4orf19 expression, age, tumor stage, gender, and grade to predict 1-, 3-, and 5-year survival probabilities. The model showed good predictive accuracy, with AUC values of 0.619 (1-year), 0.659 (3-year), and 0.630 (5-year). The calibration curves demonstrated excellent agreement between predicted and observed values. Furthermore, ROC analysis revealed that the model significantly outperformed any individual clinical parameter (Fig. 2E-H). These results establish C4orf19 as a clinically actionable prognostic biomarker in HNSCC.

### The biological function of C4orf19 in HNSCC

To investigate whether C4orf19 regulates tumor progression by modulating HNSCC cell proliferation, we first evaluated C4orf19 expression in Cal-27 and SCC-9 cell lines. SCC-9 cells showing lower baseline expression were selected to establish C4orf19-overexpressing models (Fig. S1A, B). Subsequently, we performed EdU assays, IF, and CCK-8 assays to assess proliferative capacity. EdU analysis revealed no significant difference in proliferation rates between C4orf19-overexpressing and control cells (Fig. S1C). IF staining showed comparable Ki-67 proliferation indices (Fig. S1D), and CCK-8 data confirmed similar cellular proliferative activity (Fig. S1E). These results indicate that C4orf19 does not significantly influence HNSCC cell proliferation. Western blot analysis showed that elevated C4orf19 expression did not alter PCNA levels in SCC-9 cells, but induced a subtle decline in BCL-2 expression (Fig. S1F). This suggests C4orf19 may exert a mild pro-apoptotic effect in HNSCC without affecting proliferative potential, though its precise mechanistic role requires further investigation.

**Fig. 3:**
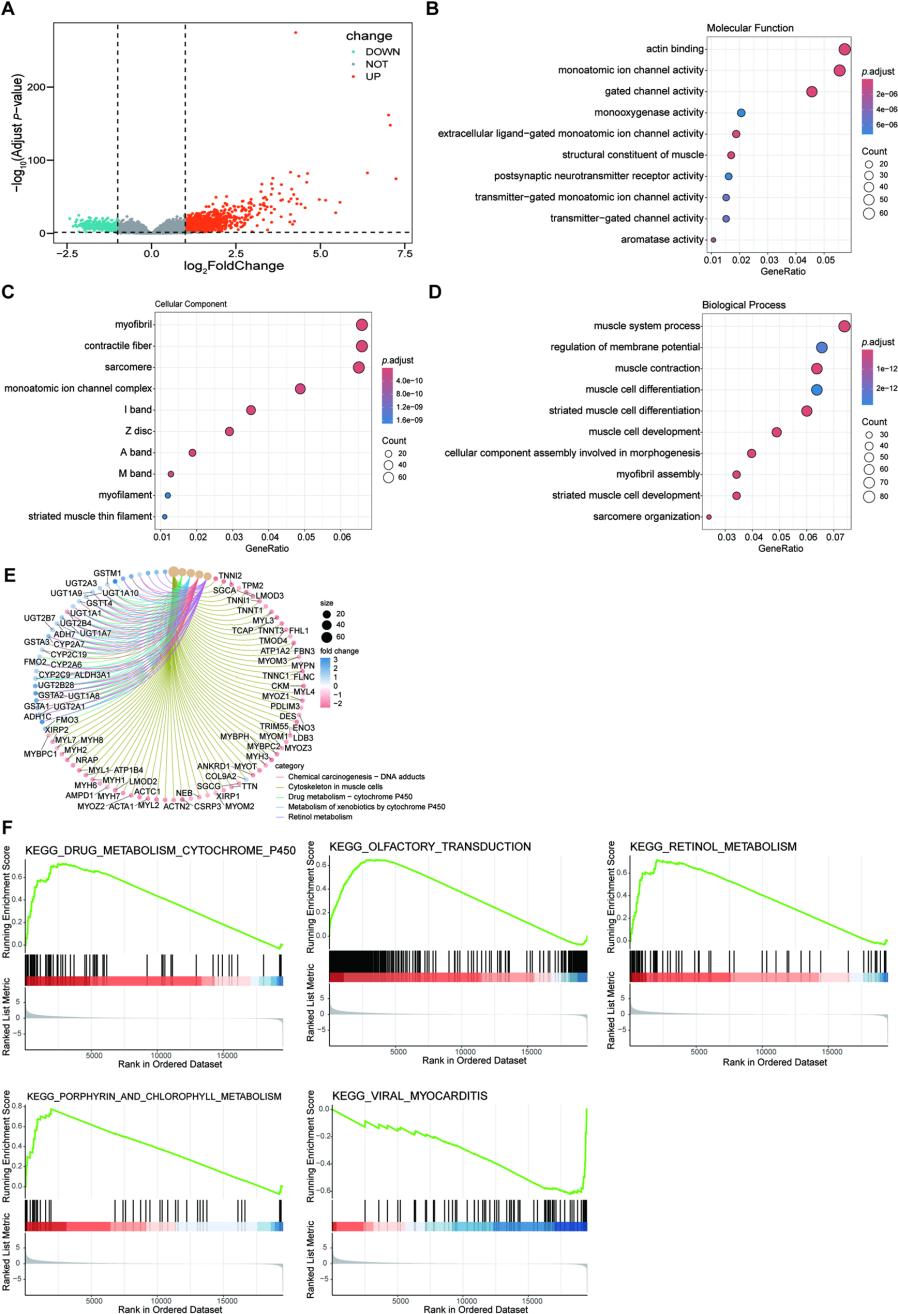
Functional enrichment analysis of DEGs grouped by C4orf19 expression. **A** Volcano plot displaying DEGs between C4orf19-high and -low groups in TCGA-HNSC dataset. Red indicates upregulated genes, blue indicates downregulated genes. **B** GO molecular function (GO-MF). **C** GO cellular component (GO-CC). **D** GO biological process (GO-BP). **E** KEGG pathway analysis. **F** GSEA

To elucidate C4orf19’s molecular mechanisms, we stratified TCGA-HNSC samples into high- and low-expression groups based on median C4orf19 expression. Differential gene expression analysis (|log₂FC| >1) identified 1, 034 upregulated and 247 downregulated genes (Fig. 3A), with hierarchical clustering visualized in a heatmap (Fig. S2). GO and KEGG enrichment analyses revealed significant enrichment in drug metabolism, cell motility, signal transduction, and muscle cytoskeleton organization (Fig. 3B-E). GSEA showed positive correlations between C4orf19 expression and cytochrome P450 (CYP450), olfactory transduction, porphyrin/chlorophyll metabolism, and retinol (vitamin A) metabolism pathways, whereas a negative correlation was observed with viral myocarditis-related pathways (Fig. 3F). These findings suggest a potential role for C4orf19 in modulating tumor drug metabolism, TME dynamics, and cancer-associated signaling cascades in HNSCC.

### C4orf19 enhances the sensitivity of HNSCC cells to 5-FU

**Fig. 4:**
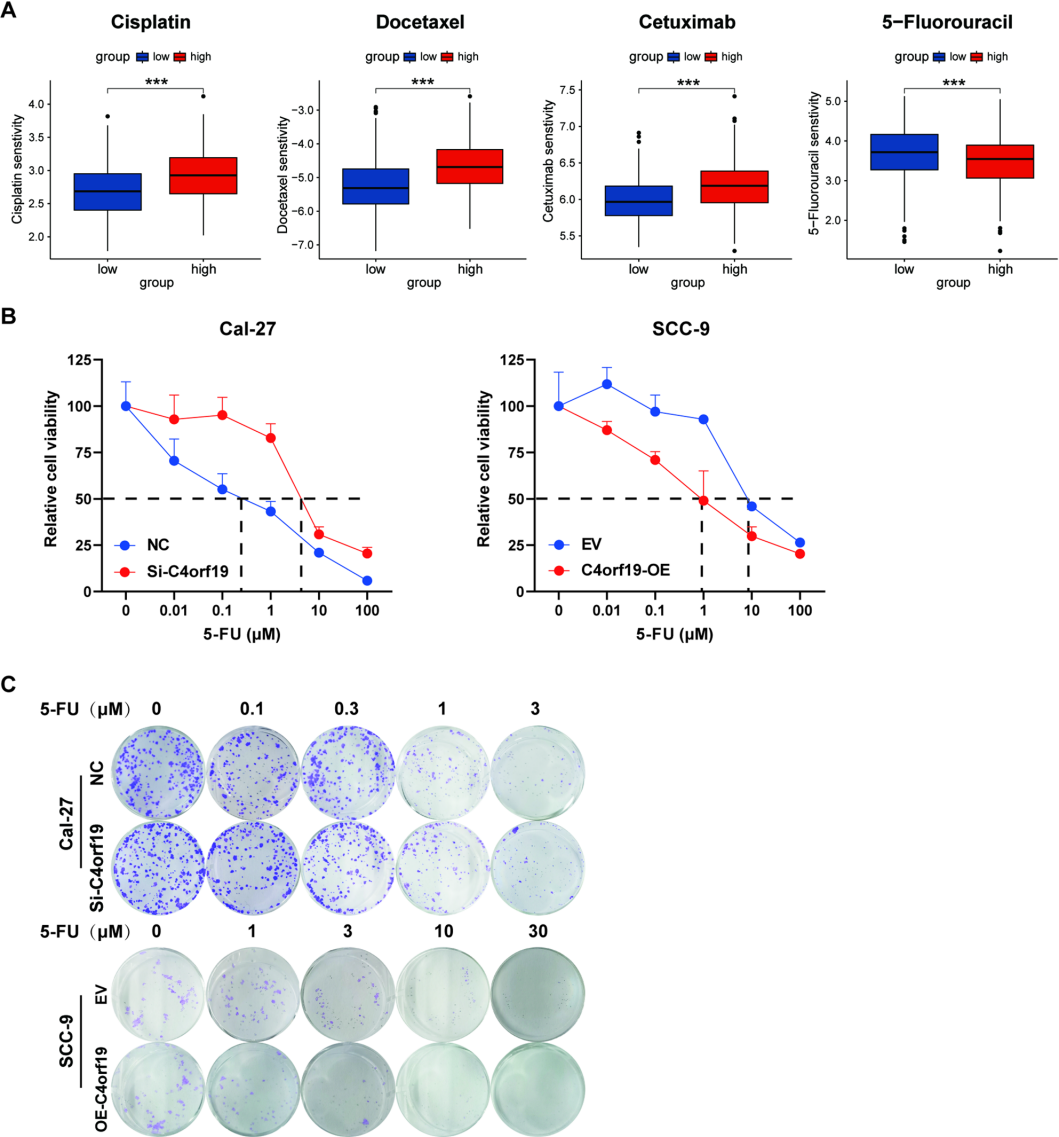
C4orf19 enhances 5-FU sensitivity in HNSCC cells. **A** Association between C4orf19 expression and drug sensitivity (IC50-like values) to cisplatin, docetaxel, cetuximab, and 5-FU in HNSCC patients. **B** CCK-8 assay measuring 5-FU IC50 in Cal-27 and SSC-9 cells with modulated C4orf19 expression. **C** Colony formation assay detecting the effect of C4orf19 on the proliferative capacity of Cal-27 and SSC-9 cells after 5-FU treatment. (*, *p* < 0.05; **, *p* < 0.01; ***, *p* < 0.001)

To investigate the role of C4orf19 in tumor drug metabolism and chemosensitivity, we analyzed responses to 367 compounds in TCGA-HNSC data, which revealed that low C4orf19 expression correlated with lower predicted IC50 values for cisplatin, docetaxel, and cetuximab but higher IC50 values for 5-FU relative to high-expression groups (Fig. 4A). We investigated the role of C4orf19 in 5-FU sensitivity using two cell models: SCC-9 with C4orf19 overexpression and Cal-27 with C4orf19 knockdown (Fig. S3). C4orf19 overexpression in SCC-9 cells reduced 5-FU IC50 (measured by CCK-8 assay), whereas knockdown in Cal-27 cells increased the IC50 (Fig. 4B). Meanwhile, knockdown experiments in Cal-27 cells revealed that C4orf19 expression did not significantly affect cisplatin IC50 values (Fig. S4). Colony formation assays further verified enhanced 5-FU sensitivity in C4orf19-overexpressing cells and resistance in knockdown models (Fig. 4C). These results demonstrate that C4orf19 serves as both a potent 5-FU chemosensitizer and a promising therapeutic target for HNSCC treatment optimization.

### The relationship between C4orf19 expression and immune checkpoint molecules

**Fig. 5:**
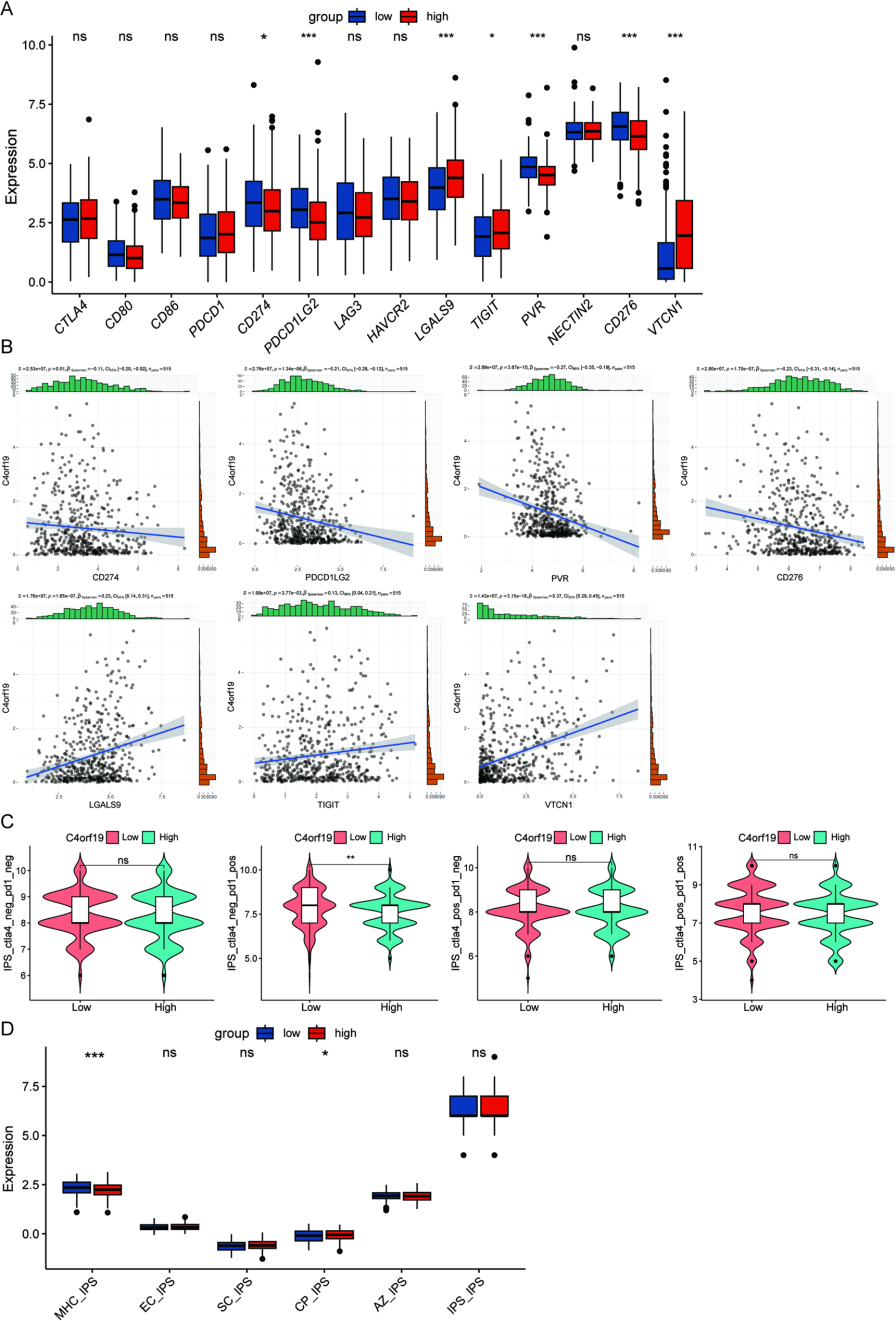
Correlation between C4orf19 expression and immune checkpoints molecules. **A** Immune checkpoint genes expression profiles in TCGA-HNSC. **B** Scatter plots demonstrating the correlation between immune checkpoint genes and C4orf19. **C** IPS analysis predicting response to immune checkpoint inhibitors. **D** Immune-related factor correlations assessed via TCIA. (*, *p* < 0.05; **, *p* < 0.01; ***, *p* < 0.001; ns, not significant)

Immunotherapy has emerged as a prominent therapeutic approach in recent years, with immune checkpoint molecules serving as key regulators of immune cell function whose inhibition can yield antitumor effects [16]. We therefore investigated correlations between C4orf19 expression and canonical immune checkpoints, revealing that LGALS9, TIGIT, and VTCN1 were upregulated in C4orf19-high groups, whereas CD274 (PD-L1), PDCD1LG2 (PD-L2), PVR (CD155), and CD276 (B7-H3) were elevated in C4orf19-low groups (Fig. 5A, B). This indicates that the expression of C4orf19 can predict the responsiveness of HNSCC patients to immunotherapy. To assess differential immunotherapy response potential, we compared Immunophenotype Scores (IPS) across expression groups. Higher IPS scores in the C4orf19-low cohorts (Fig. 5C) suggests that immunotherapy may be more effective for this type of patients. Analysis via The Cancer Immunome Atlas (TCIA) further demonstrated that C4orf19-low patients exhibited enhanced MHC-associated immunogenicity scores relative to the high-expression groups, though immunomodulator scores showed no significant intergroup differences (Fig. 5D). Collectively, these findings indicate that C4orf19 downregulation correlates with heightened immunoreactivity in HNSCC.

### The impact of C4orf19 expression on immune cell infiltration within the tumor microenvironment

**Fig. 6:**
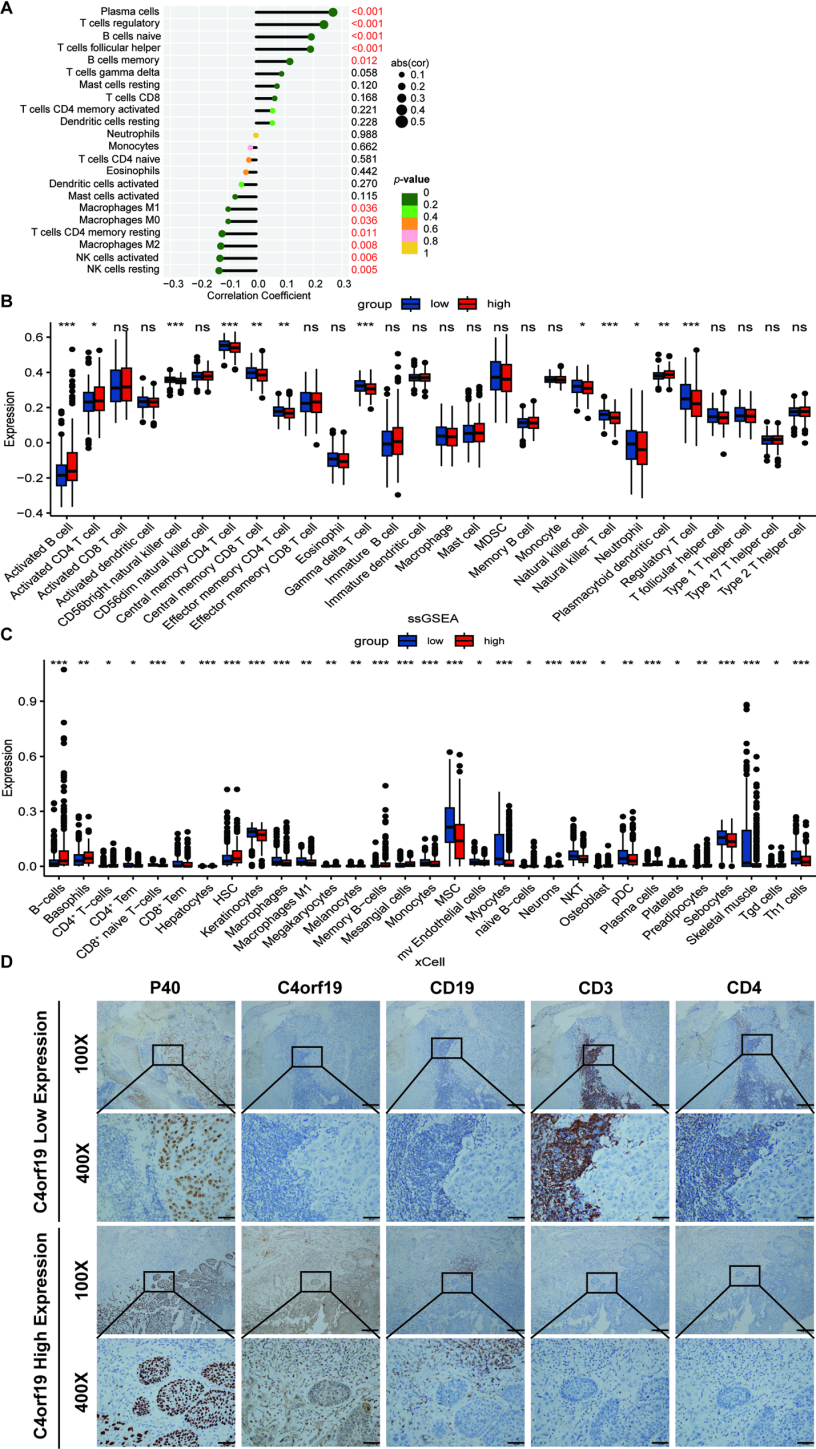
C4orf19 expression alters the TME in HNSCC. **A** Immune cell infiltration levels quantified by CIBERSORT in HNSCC patients. **B** Relative abundance of immune cell types assessed via ssGSEA in HNSCC TME. **C** Immune and stromal cell composition evaluated using Xcell algorithm. **D** IHC analysis of C4orf19 effects on T-cell (CD3^+^, CD4^+^) and B-cell (CD19^+^) infiltration in HNSCC tumor tissues (P40^+^). Scale bars: 100 μm, 20 μm

Given that immune cell infiltration within the TME influences cancer progression and patient prognosis, we investigated the relationship between C4orf19 expression and TME remodeling in HNSCC. CIBERSORT analysis of immune infiltrates revealed distinct patterns: C4orf19-high tumors exhibited increased infiltration of plasma cells, regulatory T cells (Tregs), naive B cells, follicular helper T cells, and memory B cells, whereas C4orf19-low tumors showed elevated levels of resting natural killer (NK) cells, activated NK cells, M2 macrophages, resting CD4^+^ memory T cells, M0 macrophages, and M1 macrophages (Fig. 6A).

Subsequent single-sample Gene Set Enrichment Analysis (ssGSEA) quantification of immune infiltrates revealed distinct TME profiles: C4orf19-high tumors exhibited elevated infiltration of activated B cells, activated CD4^+^ T cells, and plasmacytoid dendritic cells, whereas C4orf19-low tumors showed increased abundance of CD56bright natural killer cells, central memory CD4^+^ T cells, central memory CD8^+^ T cells, effector memory CD4^+^ T cells, γδ T cells, natural killer cells, natural killer T cells, neutrophils, and regulatory T cells (Fig. 6B).

Xcell analysis of immune cell infiltration revealed significant correlations with C4orf19 expression levels: B cells, basophils, CD4^+^ T cells, hematopoietic stem cells (HSC), memory B cells, mesangial cells, plasma cells, and γδ T cells exhibited positive correlations, whereas CD4^+^ effector memory T cells (CD4^+^ Tem), CD8^+^ effector memory T cells (CD8^+^ Tem), keratinocytes, macrophages, M1 macrophages, monocytes, mesenchymal stem cells (MSC), microvascular endothelial cells, myocytes, natural killer T cells (NKT), plasmacytoid dendritic cells (pDC), sebocytes, skeletal muscle cells, and Th1 cells showed negative correlations (Fig. 6C).

To investigate the functional impact of C4orf19 on the HNSCC TME, we performed IHC analysis of clinical specimens. Results demonstrated that low C4orf19 expression correlated with decreased abundance of CD19^+^ B lymphocytes but increased infiltration of CD3^+^ and CD4^+^ T lymphocytes within peri-tumoral regions. Conversely, elevated C4orf19 expression exhibited inverse infiltration patterns (Fig. 6D). These findings indicate that C4orf19 modulates immune cell infiltration - specifically B lymphocytes and CD4^+^ T cells - in HNSCC, suggesting its expression dynamics may reprogram the tumor immune landscape.

### The relationship between C4orf19 expression and tumor mutational burden in HNSCC

Studies indicate that high TMB correlates with increased neoantigen production. To explore the relationship between C4orf19 expression and TMB in HNSCC patients, we analyzed TCGA-HNSC data. The most prevalent mutation type in this cohort was missense mutation, primarily driven by single nucleotide polymorphisms (SNPs), with C > T transitions being the dominant single nucleotide variant (SNV). The median somatic mutation count per patient was 87, and the top five most frequently mutated genes were *TP53* (69%), *TTN* (37%), *FAT1* (21%), CDKN2A (20%) and *CSMD3* (18%) (Fig. S5).

We subsequently stratified the TCGA-HNSC cohort into C4orf19-low and C4orf19-high groups based on median expression. The C4orf19-low group exhibited higher somatic mutation frequencies in *TP53* (75%), *TTN* (31%), *CDKN2A* (25%), *FAT1* (22%), and *NOTCH1* (19%), while the C4orf19-high group showed elevated frequencies in *TP53* (65%), *TTN* (43%), *MUC16* (21%), *CSMD3* (21%), and *FAT1* (21%). Comparative analysis revealed that among these top five genes, *TTN*, *CDKN2A*, and *NOTCH1* demonstrated lower mutation rates in the C4orf19-low versus -high group (*p* < 0.05). Notably, *TP53* mutation frequency was significantly elevated in the C4orf19-low cohort relative to both the overall HNSCC cohort and the C4orf19-high group (*p* < 0.001), suggesting C4orf19 expression influences *TP53* mutational status (Fig. S6A, B). Given that *TP53* mutations serve as an established predictor of TMB, the inverse association between C4orf19 expression and *TP53* alterations indicates C4orf19 may reflect TMB levels in HNSCC.

## Discussion

HNSCC is a highly prevalent malignant tumor worldwide [17]. Despite diverse comprehensive treatment options, patients frequently contend with tumor recurrence, metastasis, and therapy resistance [18]. Consequently, there is a pressing need to identify novel therapeutic targets and diagnostic biomarkers to overcome this clinical bottleneck. To date, C4orf19’s function in various malignancies remains incompletely elucidated. In clear cell renal cell carcinoma, C4orf19 expression is downregulated [13], while it suppresses proliferation in triple-negative breast cancer and [12], in colon adenocarcinoma, exerts this effect by competitively binding Keap1 with TRIM25 to inhibit the USP17/Elk-1/CDK6 axis [14]. Using bioinformatics and IHC, we found that C4orf19 was downregulated in HNSCC and that its low expression correlated significantly with poor prognosis. Its potential as a prognostic marker was assessed using a nomogram. Nevertheless, the specific regulatory role of C4orf19 in HNSCC pathogenesis remains unclear, and its therapeutic potential requires further validation.

Our experiments showed that C4orf19 does not directly regulate HNSCC cell proliferation or anti-apoptosis. To elucidate its functional mechanism, we conducted a comprehensive differential gene expression analysis, which implicated C4orf19 in modulating CYP450-mediated metabolic pathways. Since CYP450 enzymes are critical for the metabolism of numerous chemotherapeutic drugs [19], we performed pharmacogenomic profiling [20], which showed that low C4orf19 expression was associated with increased sensitivity to cisplatin, docetaxel, and cetuximab, but reduced sensitivity to 5-FU. *In* *vitro* assays corroborated that high C4orf19 expression enhances 5-FU sensitivity. Given this discrepancy, we propose a model wherein C4orf19-mediated 5-FU sensitization occurs not through direct growth inhibition but via altered drug metabolism. As 5-FU is an antimetabolite [21] whose activity is modulated by metabolic enzymes in the TME [22], we hypothesize that C4orf19 activates CYP450 signaling, accelerating 5-FU metabolic activation and/or reducing its clearance. This increases intracellular drug exposure, leading to enhanced cytotoxic effects. Consequently, high C4orf19 expression could serve as a biomarker for 5-FU treatment selection in HNSCC.

Immune checkpoint inhibitors (ICIs) represent a standard treatment for recurrent or metastatic HNSCC [23]; however, the identification of robust predictive biomarkers remains a challenge [24, 25]. This study identifies C4orf19 as a novel modulator of the tumor immune microenvironment and a potential biomarker for immunotherapy. Low C4orf19 expression was associated with upregulation of multiple immune checkpoint molecules (including CD274/PD-L1 and PDCD1LG2/PD-L2) [26–28] and an elevated MHC-related immunogenicity score [29–31]. This suggests that C4orf19 may serve as a biomarker to reflect the immune activity of tumors and, to a certain extent, predict the efficacy of immunotherapy. Analysis of the TME revealed increased infiltration of macrophages, NK cells, and CD4^+^ T cells, coupled with a reduction in B cells, in C4orf19-low tumors. IHC validation confirmed this distinctive immune landscape, showing increased CD3^+^/CD4^+^ T lymphocytes and decreased CD19^+^ B lymphocytes. Paradoxically, this specific immune contexture may underlie the predicted superior response to ICIs in this subgroup [32–34]. Furthermore, low C4orf19 expression was linked to a higher TMB, which was inferred from the increased TP53 mutation rate. TMB was a known predictive factor for the efficacy of ICI therapy [35–38]. Collectively, these findings suggest that patients with low C4orf19 expression may be optimal candidates for immunotherapy. However, since all the above studies were based on bioinformatics predictions of previous data, it remains to be determined through extensive clinical research whether C4orf19 can serve as a marker for guiding clinical immunotherapy.

In conclusion, our study demonstrates for the first time that C4orf19 expression exhibits significant complexity and clinical guiding value in HNSCC, serving as a multifaceted biomarker for predicting prognosis and guiding treatment strategies. Bioinformatics analysis reveals that C4orf19 plays a crucial dual role in HNSCC. Its high expression is significantly associated with a favorable prognosis for patients and a higher sensitivity to 5-FU chemotherapy. Conversely, low expression of C4orf19 indicates that patients may have a better clinical response to other standard therapies such as cisplatin, docetaxel, cetuximab, and immune checkpoint inhibitors. Although cell experiments have preliminarily confirmed that C4orf19 can enhance the sensitivity of 5-FU, its application value as a clinical biomarker still needs to be verified through large-scale clinical studies. In summary, C4orf19 is expected to become a key decision-making marker guiding individualized treatment for HNSCC. By assessing its expression level, it may enable the precise selection of patient groups most suitable for chemotherapy or immunotherapy, thereby promoting the development of precise medical care for HNSCC.

## Supplementary Information

Supplementary Material 1.

Supplementary Material 2.

## Data Availability

The data supporting this study’s findings are available from the corresponding author upon reasonable request.

## Abbreviations

5-FU: 5-fluorouracil
AUC: Area under the curve
ICIs: Checkpoint inhibitors
C4orf19: Chromosome 4 Open Reading Frame 19
GO: Gene Ontology
GSEA: Gene set enrichment analysis
HNSCC: Head and neck squamous cell carcinoma
HPV: Human papillomavirus
IPS: Immunophenoscore
KM: Kaplan-Meier
KEGG: Kyoto Encyclopedia of Genes and Genomes
OS: Overall survival
ROC: Receiver operating characteristic
SNPs: Single nucleotide polymorphisms
SNV: Single nucleotide variant
TCGA: The Cancer Genome Atlas
TMB: Tumor mutation burden
TME: Tumor microenvironment
