# Supplementary material for: Multi-omics characterization of C4orf19 in HNSCC: constructing prognostic signatures for immunotherapy and chemotherapy response prediction
Source: BMC Cancer. 2026 Jan 30;26:309. doi: 10.1186/s12885-026-15633-y (PMC12947368; doi:10.1186/s12885-026-15633-y)
Supplement: Supplementary file 1 — Supplementary Material 1. [file 12885_2026_15633_MOESM1_ESM.docx]

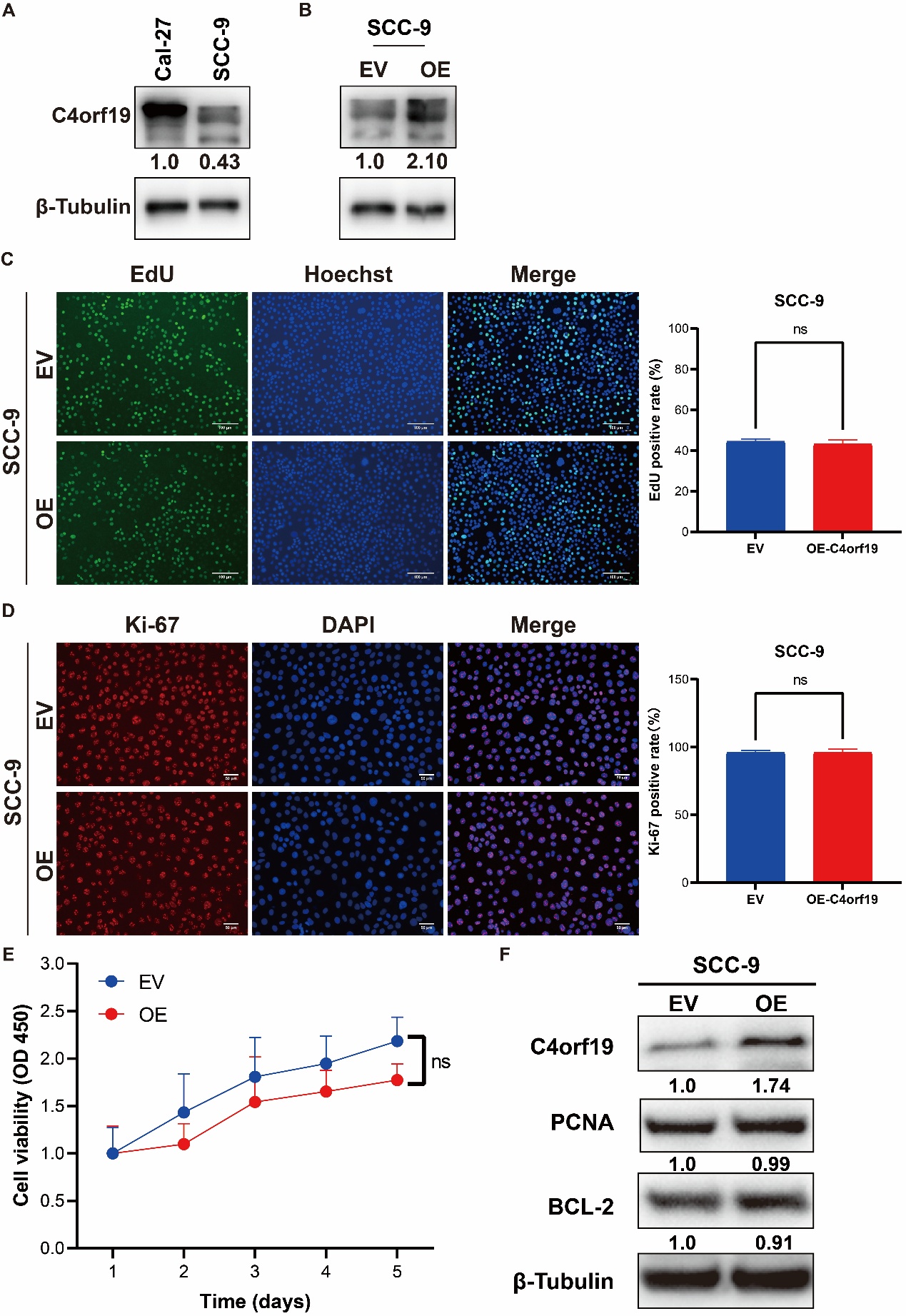


Fig S1. Establishment of C4orf19-overexpressing cell lines and its impact on HNSCC cell proliferation

(A) Western blot analysis of C4orf19 expression in HNSCC cell lines Cal-27 and SCC-9. (B) Western blot confirming C4orf19 overexpression in SCC-9 cells. (C) EdU assay assessing the effect of C4orf19 expression on SCC-9 proliferation, with representative images and quantification. Scale bar: 100 μm. (D) IF staining of Ki-67 to evaluate proliferation in SCC-9 cells with altered C4orf19 expression, along with representative images and quantification. Scale bar: 50 μm. (E) CCK-8 assay measuring SCC-9 cell proliferation under different C4orf19 expression levels. (F) Western blot analysis of proliferation marker PCNA and anti-apoptotic protein BCL-2 in SCC-9 cells with modulated C4orf19 expression. (*, *p* < 0.05; **, *p* < 0.01; ***, *p* < 0.001; ns, not significant).


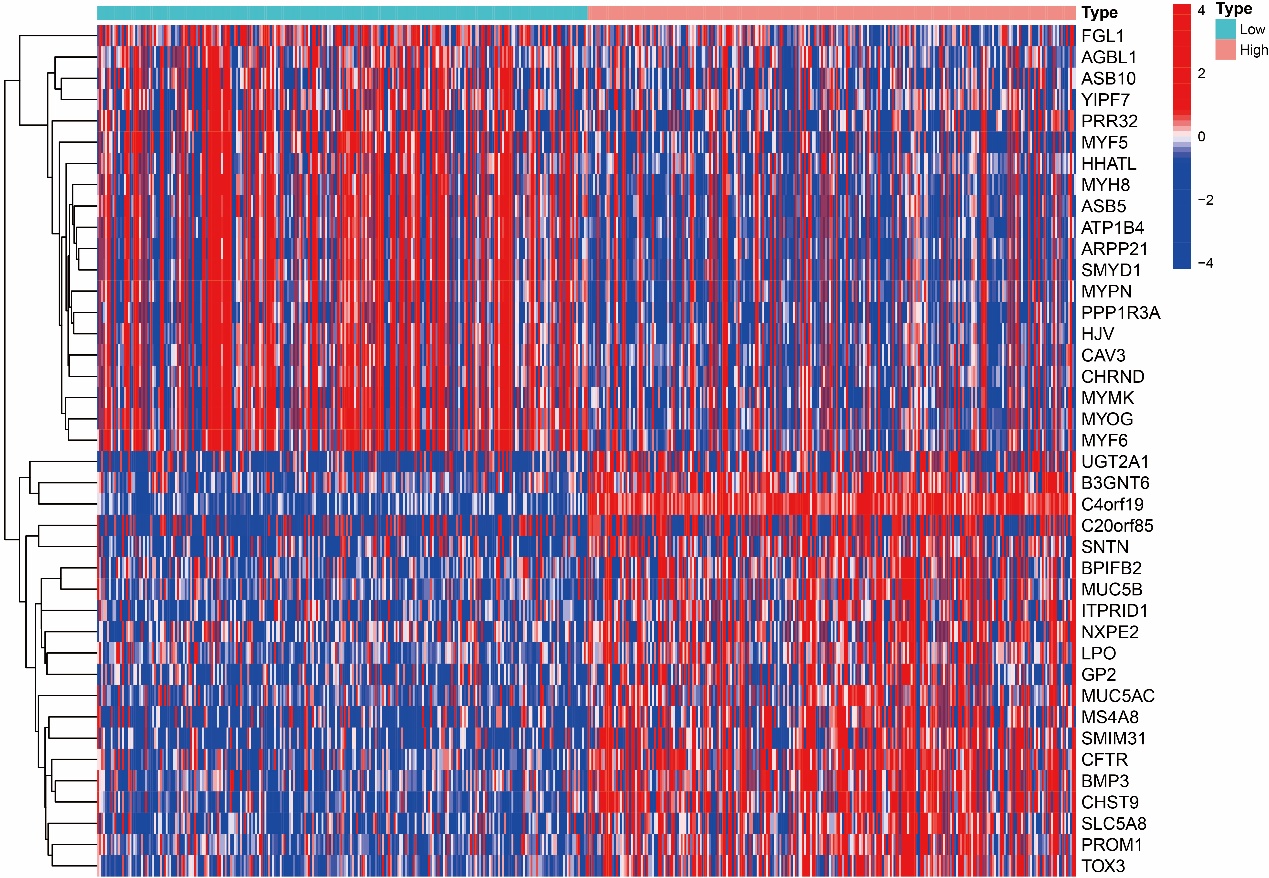


Fig S2. Identification of DEGs based on C4orf19 expression levels

Heatmap visualization of DEGs among different C4orf19 expression groups.


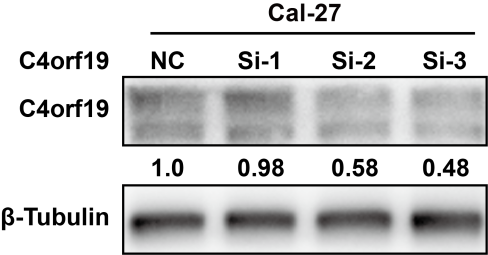


Fig S3. Establishment of C4orf19-knockdown cell lines

Western blot analysis confirming C4orf19 knockdown efficiency in Cal-27 cells.


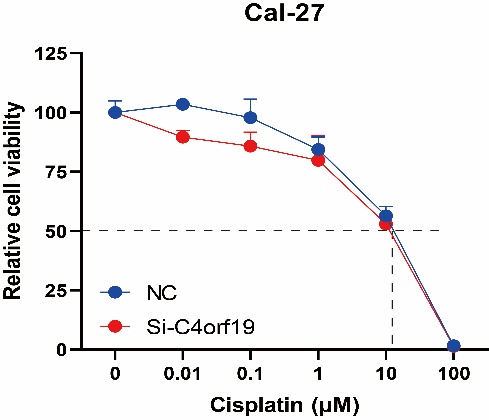


Fig S4. The effect of C4orf19 knockdown on the cisplatin sensitivity of HNSCC cells

CCK-8 assay to measure cisplatin IC50 values in Cal-27 cells with different C4orf19 expression levels.


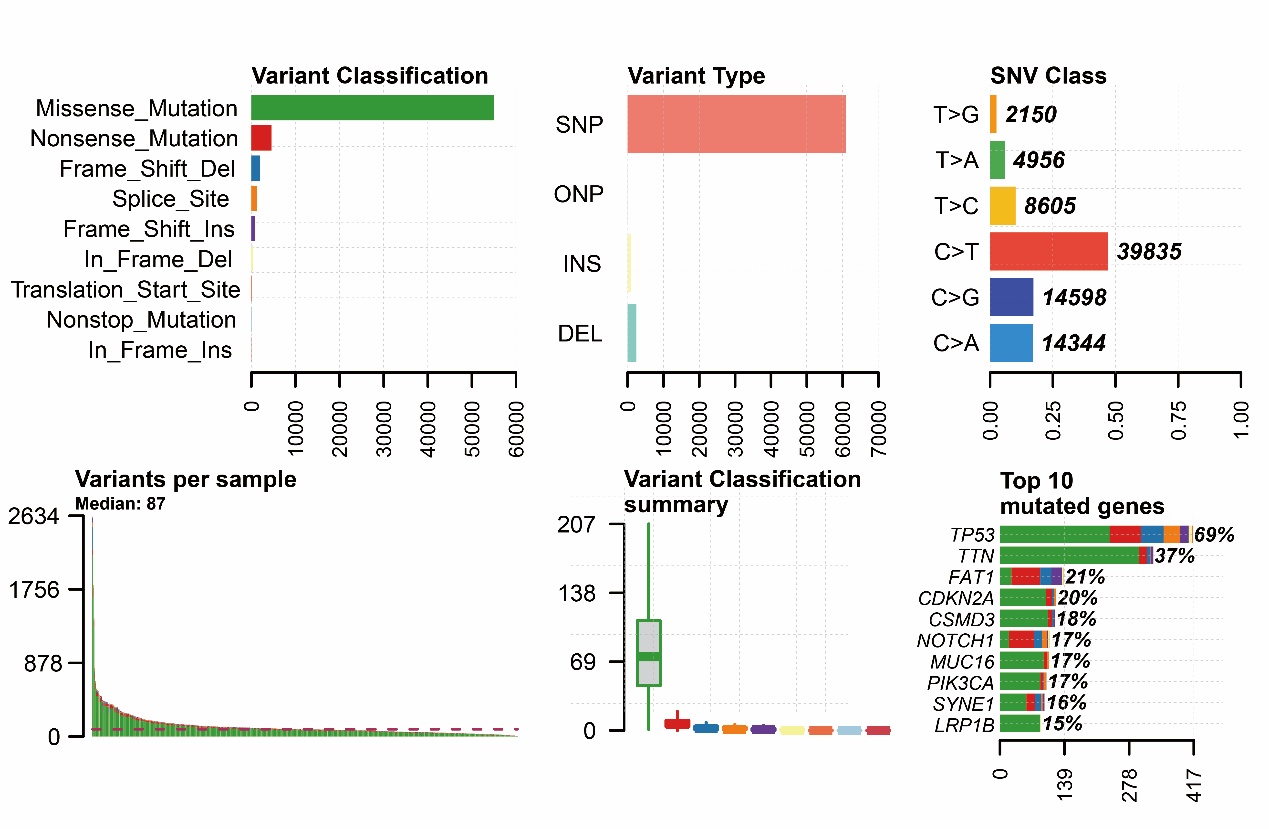


Fig S5. Somatic Gene Mutation Landscape in HNSCC Patients

Waterfall plot depicting the somatic mutation profile of HNSCC patients from the TCGA-HNSC cohort.


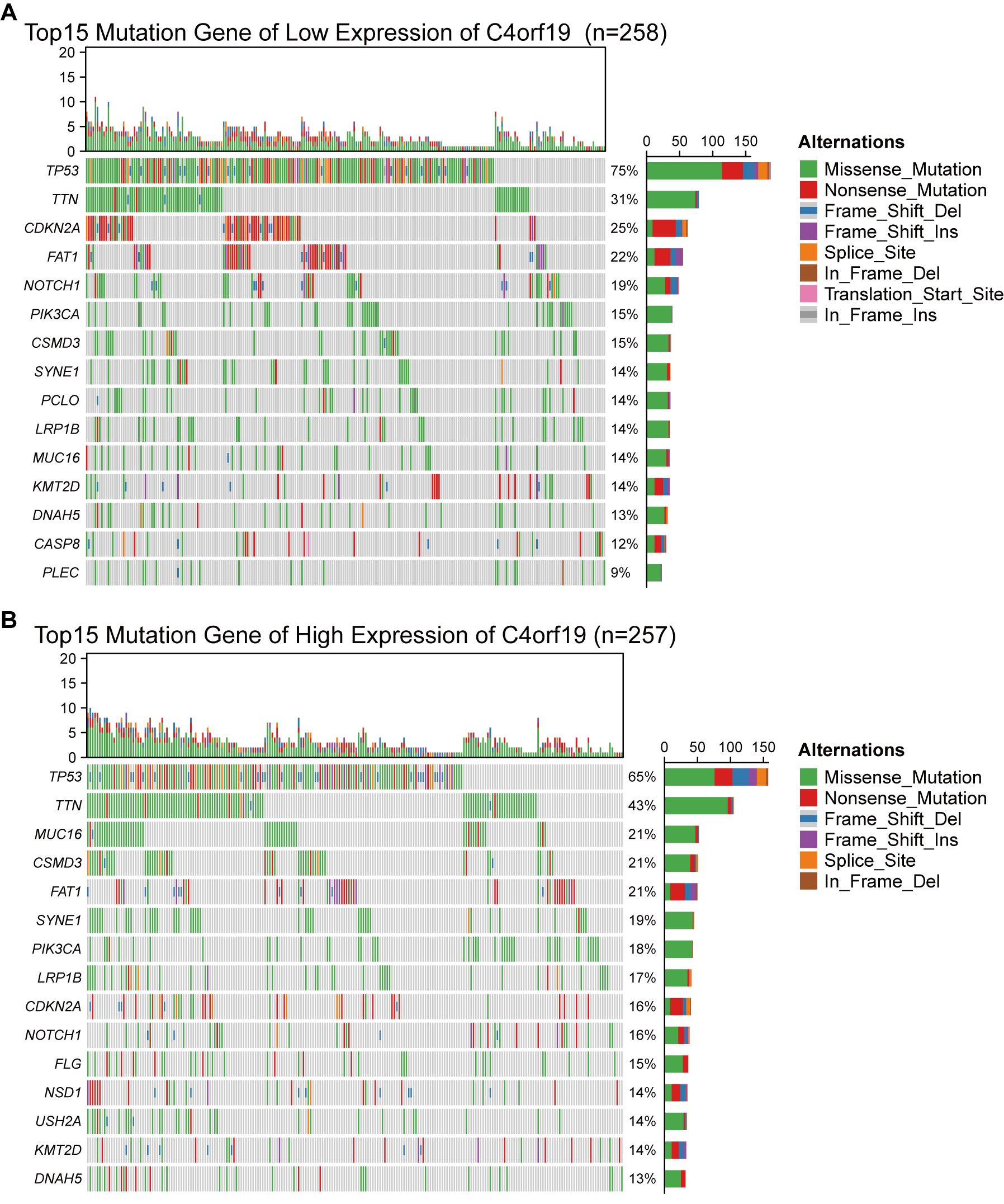
Fig S6. Comparative analysis of somatic mutations by C4orf19 expression levels

Somatic mutation profiles in the TCGA-HNSC cohort stratified by C4orf19 expression, (A) Low C4orf19 expression group. (B) High C4orf19 expression group.

**Supplementary Table 1. List of antibodies for IHC, IF, WB analyses.**

| Name | Source | Identifier | IHC Dilution | IF Dilution | WB Dilution |
| --- | --- | --- | --- | --- | --- |
| C4orf19 | Invitrogen | PA5-34718 | 1:100 | N/A | 1:1000 |
| β-Tubulin | Bioworld | AP0064 | N/A | N/A | 1:1000 |
| PCNA | Cell Signaling Technology | 13110S | N/A | N/A | 1:1000 |
| BCL2 | Cell Signaling Technology | 2870S | N/A | N/A | 1:1000 |
| Ki-67 | Huabio | Ha721115 | N/A | 1:200 | N/A |
| CD3 | ZSGB-Bio | ZM-0417 | 1:1 | N/A | N/A |
| CD4 | MXB | RMA-0620 | 1:1 | N/A | N/A |
| CD19 | MXB | MAB-0705 | 1:1 | N/A | N/A |
| P40 | MXB | RMA-0815 | 1:1 | N/A | N/A |

**Supplementary Table 2. Probes for siRNAs**

| Gene | Sense (5’-3’) | Antisense (5’-3’) |
| --- | --- | --- |
| SiC4orf19_1 | GAAGACGACACUGAUAAAUTT | AUUUAUCAGUGUCGUCUUCTT |
| SiC4orf19_2 | GGCCAGUACUGCAAAUACUTT | AGUAUUUGCAGUACUGGCCTT |
| SiC4orf19_3 | GGACCCAAGUCAUGAGAAATT | UUUCUCAUGACUUGGGUCCTT |
| NC SiRNA | UUCUCCGAACGUGUCACGUTT | ACGUGACACGUUCGGAGAATT |
