# Supplementary material for: Multi-omics characterization of C4orf19 in HNSCC: constructing prognostic signatures for immunotherapy and chemotherapy response prediction
Source: BMC Cancer. 2026 Jan 30;26:309. doi: 10.1186/s12885-026-15633-y (PMC12947368; doi:10.1186/s12885-026-15633-y)

Fig S1A

β-Tubulin

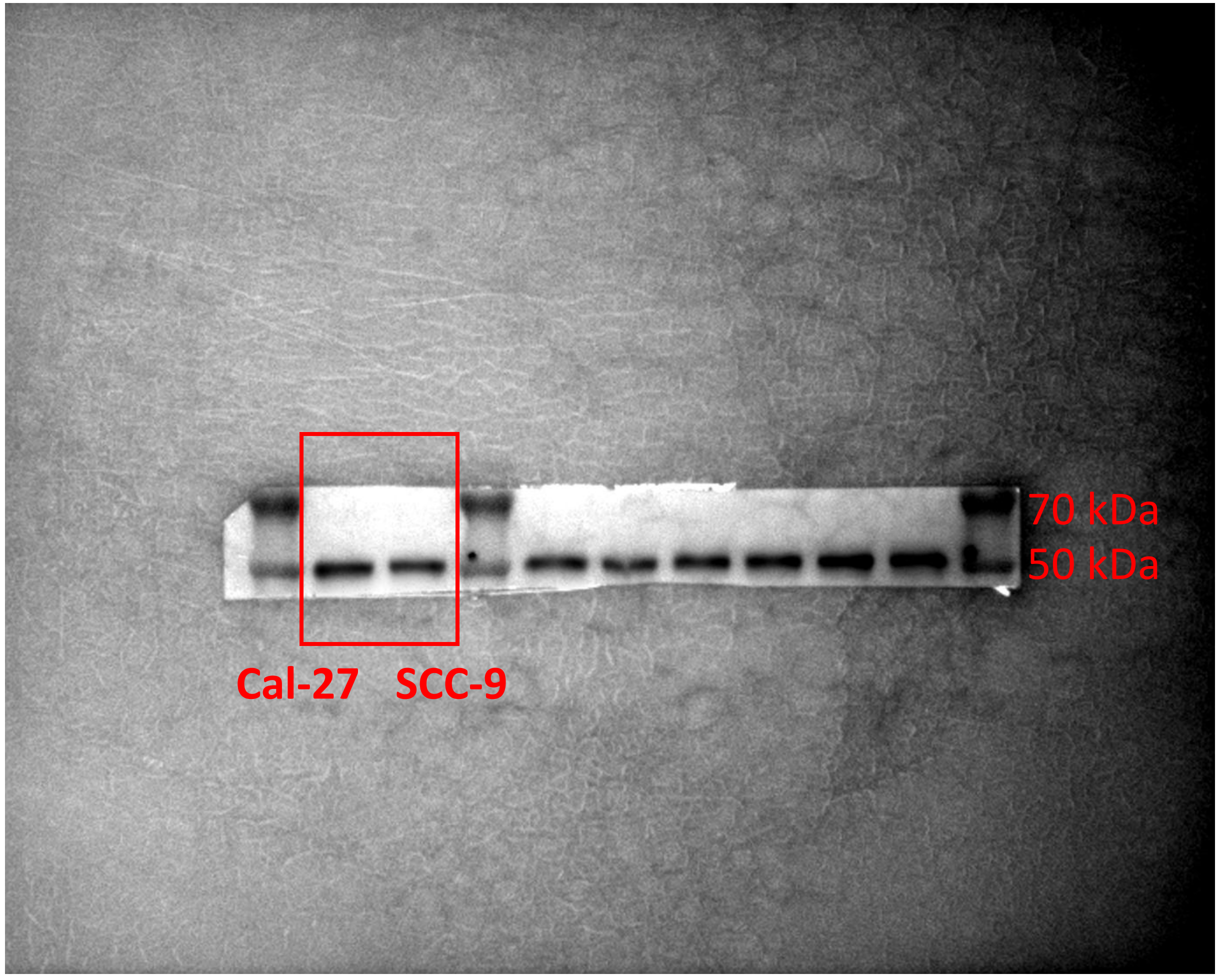

C4orf19

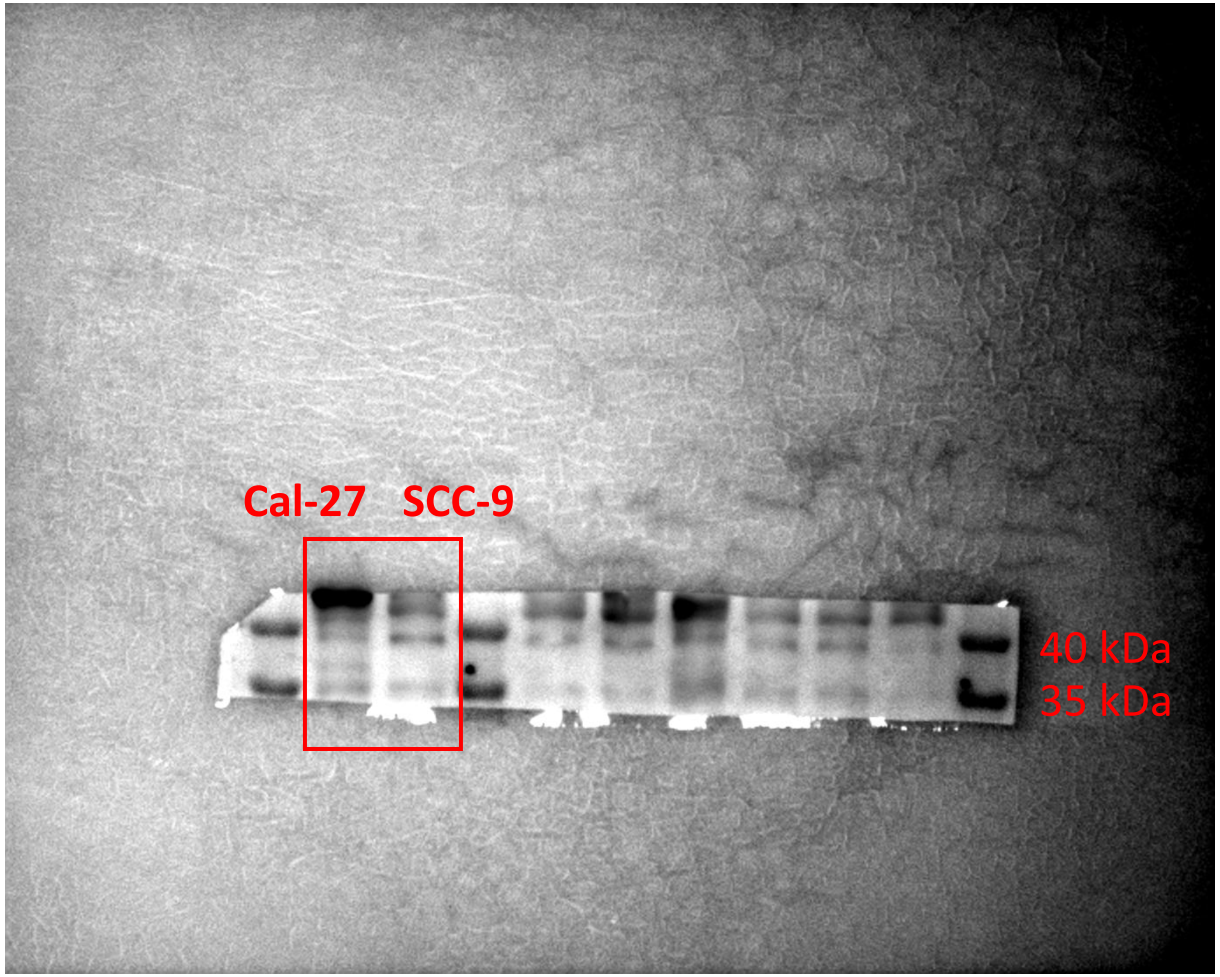

Fig S1B

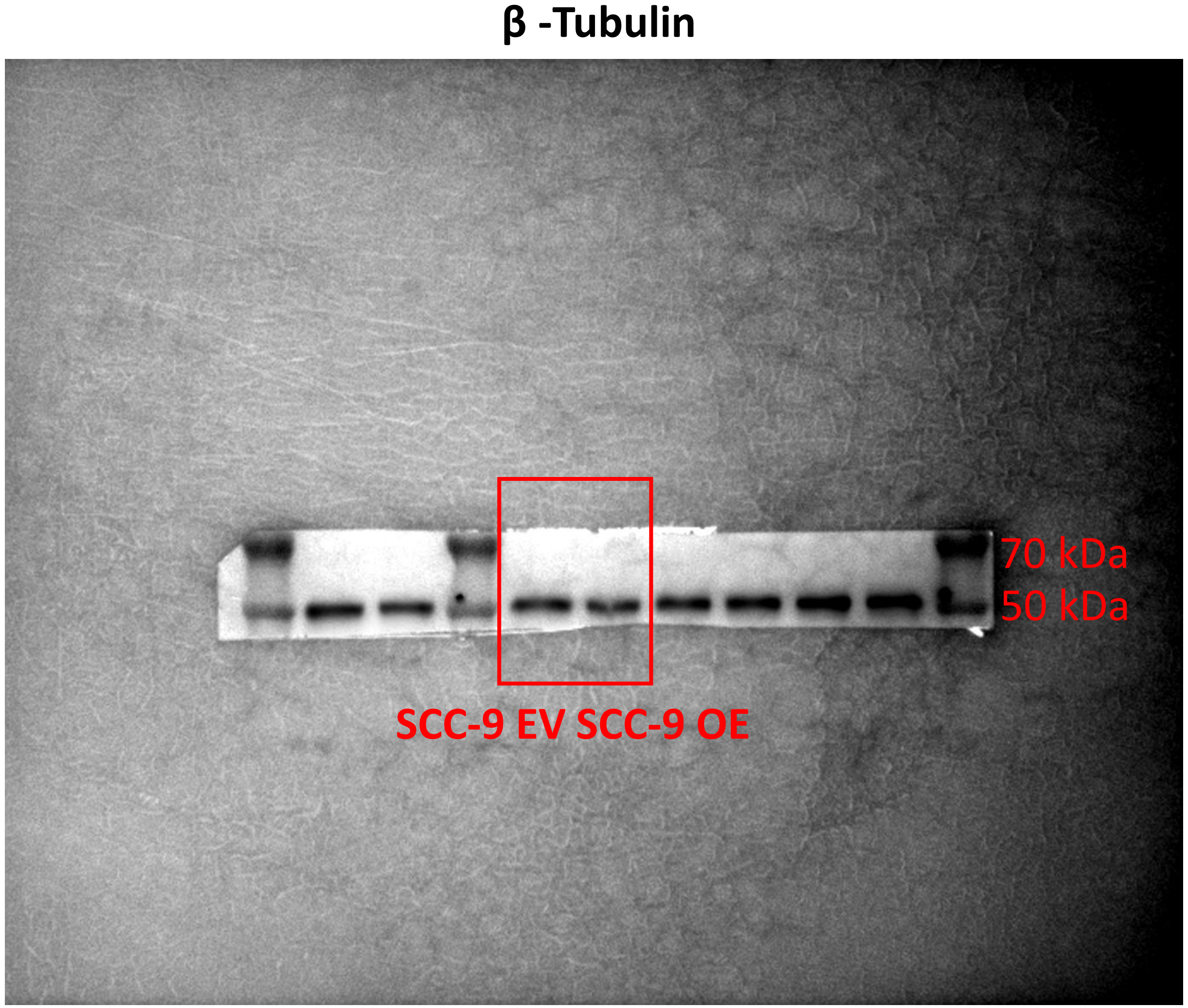
β -Tubulin
70 kDa
50 kDa
SCC-9 EV SCC-9 OE

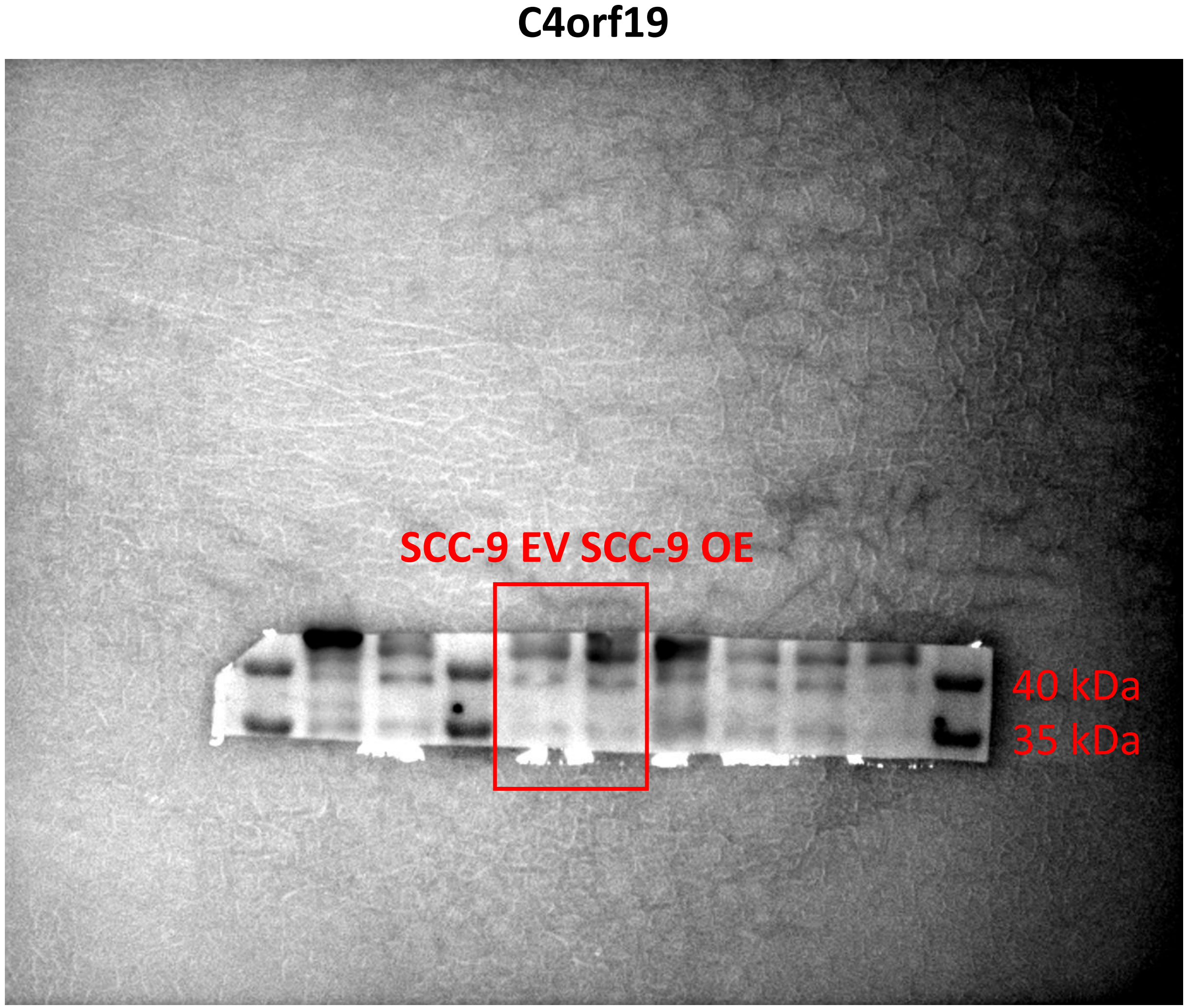
C4orf19
SCC-9 EV SCC-9 OE
40 kDa
35 kDa

Fig S1F

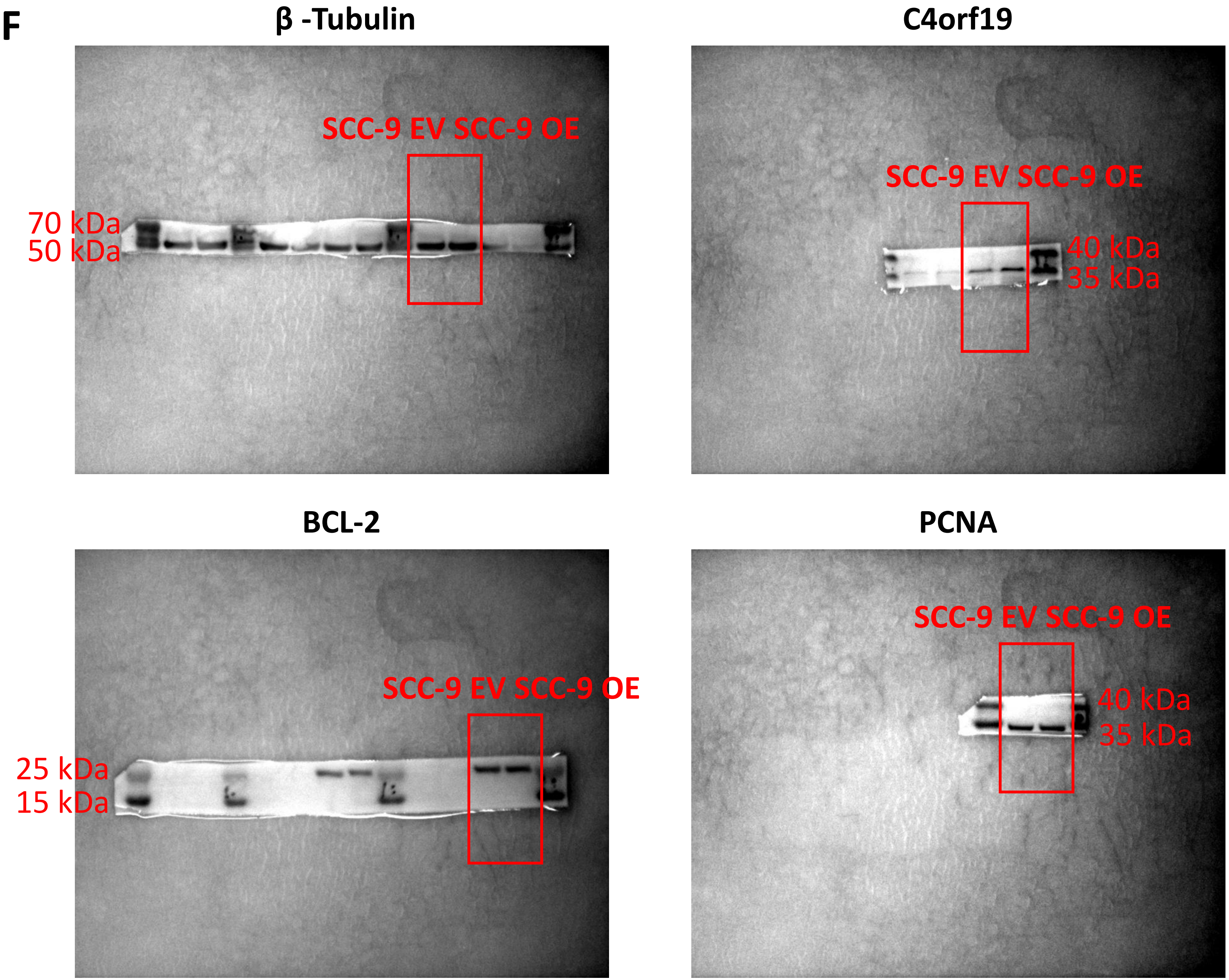
β -Tubulin
SCC-9 EV SCC-9 OE
70 kDa
50 kDa
C4orf19
SCC-9 EV SCC-9 OE
40 kDa
35 kDa
BCL-2
SCC-9 EV SCC-9 OE
25 kDa
15 kDa
PCNA
SCC-9 EV SCC-9 OE
40 kDa
35 kDa

Fig S3

β -Tubulin

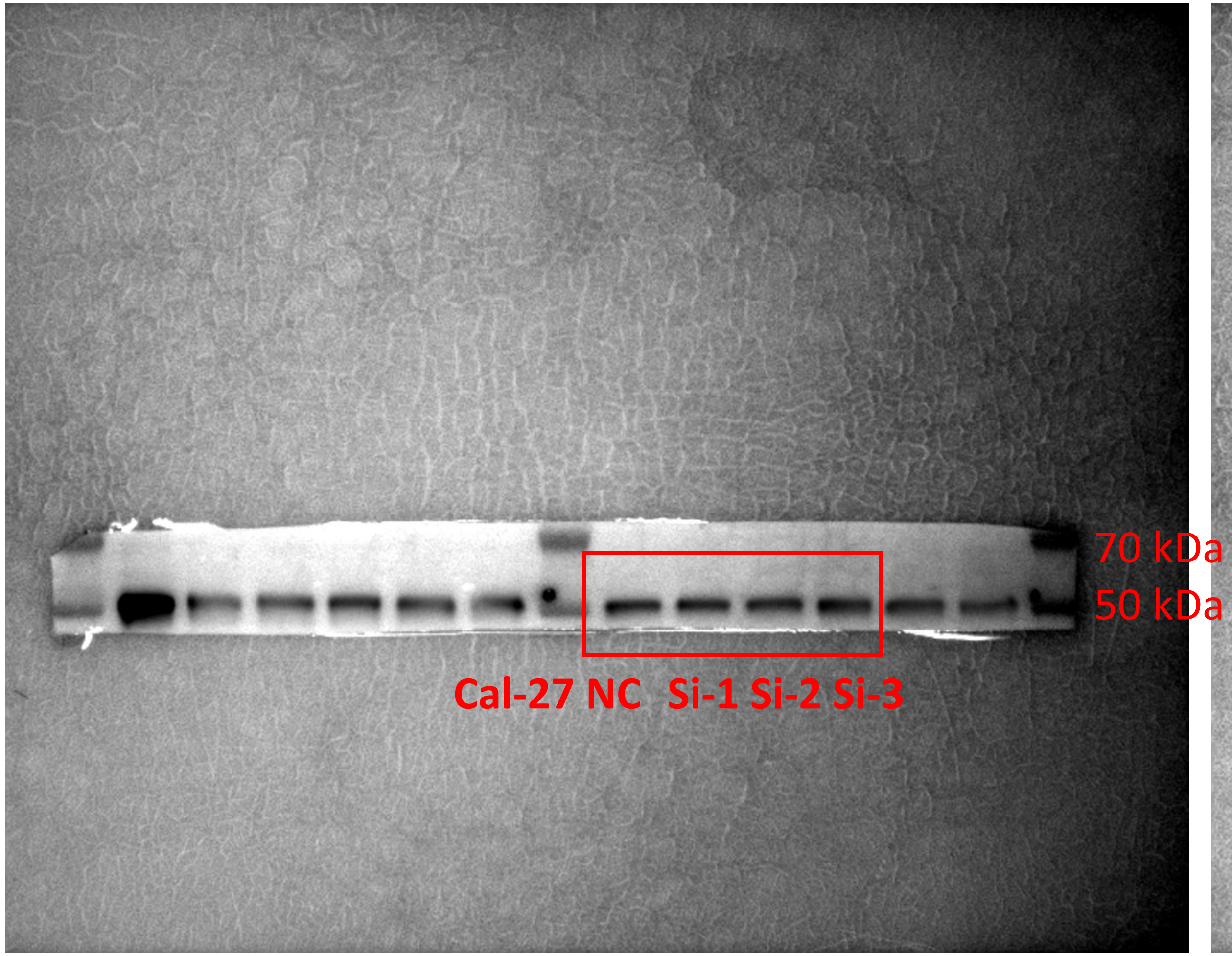

C4orf19

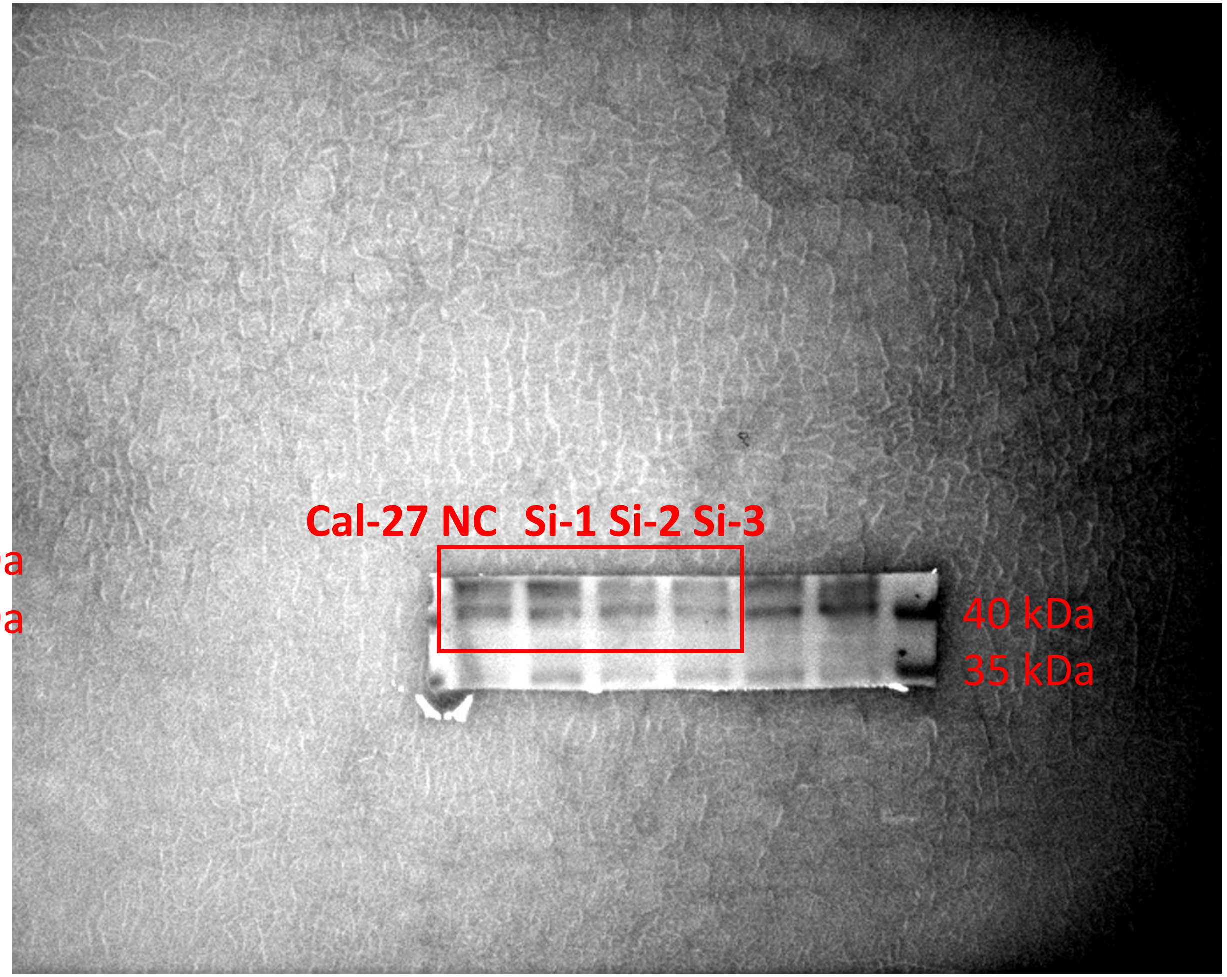

Supplement: Supplementary file 2 — Supplementary Material 2. [file 12885_2026_15633_MOESM2_ESM.pdf]
